# Clinical Utility of Transcranial Magnetic Stimulation (TMS) in the Presurgical Evaluation of Motor, Speech, and Language Functions in Young Children With Refractory Epilepsy or Brain Tumor: Preliminary Evidence

**DOI:** 10.3389/fneur.2021.650830

**Published:** 2021-05-19

**Authors:** Shalini Narayana, Savannah K. Gibbs, Stephen P. Fulton, Amy Lee McGregor, Basanagoud Mudigoudar, Sarah E. Weatherspoon, Frederick A. Boop, James W. Wheless

**Affiliations:** ^1^Division of Pediatric Neurology, Department of Pediatrics, University of Tennessee Health Science Center, Memphis, TN, United States; ^2^Le Bonheur Children's Hospital, The Neuroscience Institute, Memphis, TN, United States; ^3^Department of Anatomy and Neurobiology, University of Tennessee Health Science Center, Memphis, TN, United States; ^4^Semmes Murphey Neurologic and Spine Institute, Memphis, TN, United States; ^5^Department of Neurosurgery, University of Tennessee Health Science Center, Memphis, TN, United States

**Keywords:** transcranial magnetic stimulation, motor mapping, language mapping, epilepsy, brain tumor, presurgical, children, speech mapping

## Abstract

Accurate presurgical mapping of motor, speech, and language cortices, while crucial for neurosurgical planning and minimizing post-operative functional deficits, is challenging in young children with neurological disease. In such children, both invasive (cortical stimulation mapping) and non-invasive functional mapping imaging methods (MEG, fMRI) have limited success, often leading to delayed surgery or adverse post-surgical outcomes. We therefore examined the clinical utility of transcranial magnetic stimulation (TMS) in young children who require functional mapping. In a retrospective chart review of TMS studies performed on children with refractory epilepsy or a brain tumor, at our institution, we identified 47 mapping sessions in 36 children 3 years of age or younger, in whom upper and lower extremity motor mapping was attempted; and 13 children 5–6 years old in whom language mapping, using a naming paradigm, was attempted. The primary hand motor cortex was identified in at least one hemisphere in 33 of 36 patients, and in both hemispheres in 27 children. In 17 children, primary leg motor cortex was also successfully identified. The language cortices in temporal regions were successfully mapped in 11 of 13 patients, and in six of them language cortices in frontal regions were also mapped, with most children (*n* = 5) showing right hemisphere dominance for expressive language. Ten children had a seizure that was consistent with their clinical semiology during or immediately following TMS, none of which required intervention or impeded completion of mapping. Using TMS, both normal motor, speech, and language developmental patterns and apparent disease induced reorganization were demonstrated in this young cohort. The successful localization of motor, speech, and language cortices in young children improved the understanding of the risk-benefit ratio prior to surgery and facilitated surgical planning aimed at preserving motor, speech, and language functions. Post-operatively, motor function was preserved or improved in nine out of 11 children who underwent surgery, as was language function in all seven children who had surgery for lesions near eloquent cortices. We provide feasibility data that TMS is a safe, reliable, and effective tool to map eloquent cortices in young children.

## Introduction

Presurgical mapping of the critical cortex in patients undergoing neurosurgery is critical in assisting surgical planning and minimizing post-operative deficits. Cortical stimulation mapping (CSM) has long been considered the “gold standard” for identifying motor and language cortices, much like intracarotid sodium amytal (Wada) testing, an invasive technique used to evaluate language dominance. The realization that CSM and Wada testing have a number of limitations (1, 2) has led to the emergence of safer non-invasive alternatives, including magnetoencephalography (MEG) (3), functional magnetic resonance imaging (fMRI) (4, 5), and more recently, transcranial magnetic stimulation (TMS) (6–10). Presently, these non-invasive methods are approved by the US Food and Drug Administration for use in presurgical functional mapping and are used alongside Wada and CSM to determine hemispheric dominance, to localize motor, speech, and language cortices in the vicinity of the lesion, and to plan the surgical approach. While these mapping methods can be readily administered in older children and adults (11–14), functional mapping in young children continues to be challenging. Neurological disorders such as tuberous sclerosis, perinatal stroke, hemimegaloencephaly, and pediatric tumors occur early in childhood and may result in refractory epilepsy. The optimal treatment for these disorders, especially in those who fail multiple antiepileptic drugs (AEDs), is often surgery. In such cases, precise mapping of brain function is paramount. However, the ability to perform functional mapping in this population, is limited, and may delay surgical treatments that could greatly improve cognitive function and the quality of life (15). In such young children, TMS could be uniquely suited to overcome challenges that other methods cannot, providing clinicians and patients with information critical to improved outcomes.

With respect to motor function, accurate localization of the motor cortex is challenging in patients under the age of 3 years, particularly if there is associated developmental delay. This is especially true for modalities like MEG and fMRI, and in such cases, mapping is typically attempted during natural sleep or sedation (16–19). Still, MEG and fMRI performed under these conditions are subject to unique limitations in this age group, including the indirect nature of motor cortex localization *via* sensorimotor tasks and the risks associated with sedation. In such situations, TMS has the advantage of directly mapping the motor cortex without the need for absolute patient cooperation (20). Since patients are not required to be still during TMS mapping, there is no need for sedation. Further, mapping using TMS is also possible in patients who are unable to perform motor tasks due to diseases such as paresis or plegia or due to behaviors such with autism or developmental delay. Therefore, TMS is singularly situated for use in non-invasively evaluating the motor system in young children under 3 years of age, including those with developmental delays, directly and without requiring sedation.

Like motor mapping in infants and toddlers, language mapping in pre-school children is often difficult. Children, particularly between 5 and 6 years old, make up one such group (21–23) in whom CSM poses significant risks (i.e., higher charge density (24) causing after-discharges and seizures) with only limited success. For instance, the success rate of CSM is reported to be under 50% in children under 10 years (age range 4.7–10 years) (25). Although fMRI and MEG modalities have gained widespread acceptance, pre-school children often are unable to cooperate with the testing demands of these procedures due to developmental delay, claustrophobia, or general anxiety. In fact, both fMRI and MEG do not yield language mapping results in ~30% of cases (19, 26). Because children in this age group are sedated during fMRI and MEG and in instances when mapping is successful, it is limited to localization of receptive language areas (27). The lack of thorough speech and language localization for this group likely prevents timely surgeries that could significantly improve cognitive function (28) and quality of life (15, 23); in other cases, surgery proceeds without a precise language map, resulting in possible post-operative speech and language deficits (29) or inadequate resection. However, when compared to fMRI and MEG, TMS has many advantages. First, TMS does not require the patient to remain still. Second, unlike MEG and fMRI where task performance is covert, TMS requires overt speech, and therefore performance can be monitored for accuracy. Third, expressive language mapping with TMS is also possible in young children who can only undergo receptive language mapping under sedation in MEG and fMRI (27). Fourth, because it is performed in the awake state, TMS language mapping can be performed across multiple sessions, or repeated for verification. Fifth, motor and language mapping with TMS are not impeded by contraindications from most intrinsic metals (unlike MEG and fMRI). Sixth, TMS results are not affected by potential signal artifacts from vascular anomalies and tumor-induced neurovascular uncoupling, as in the case of fMRI (30). Finally, the use of MRI guidance to visualize the cortical surface and accurately position TMS, also termed navigated TMS, has greatly facilitated the use of TMS in children. Henceforth in the manuscript, TMS refers to delivery of TMS under MRI guidance. For these reasons, in young children and other patients in whom alternative methods fail, functional mapping with TMS is especially promising.

At our institution, functional mapping with TMS is successful in nearly 90% of patients in whom MEG and fMRI are not able to provide a motor and/or language map. We have previously reported a small case-series of six children under the age of 3 years who underwent TMS motor mapping at our institution (20) and on the utility of TMS motor mapping in an infant with cortical dysplasia (31). We have also reported a case study of a 4-year 11-month-old child who underwent successful TMS language mapping (32). In this paper we aim to further assess whether reliable motor maps can be derived in very young children with neurological disorders and evaluate the safety and tolerability of TMS in a larger cohort of children younger than 3 years. Furthermore, we report here our experience of mapping speech and language functions in 5- and 6-year-old preschool children, an age group in whom both invasive and non-invasive methods are often unsuccessful. We describe challenges in mapping this population and strategies that have facilitated successful mapping. We also present post-surgical motor and language outcomes in children when available.

## Methods

### Patients

We performed a retrospective chart review of TMS motor and language mapping studies attempted between January 2013 and September 2020 at Le Bonheur Children's Hospital, Memphis TN. The institutional review boards at the University of Tennessee Health Science Center and Le Bonheur Children's Hospital approved the retrospective chart review. We identified 47 motor mapping sessions performed on 36 children under the age of 3 years. Six children were mapped twice, one child was mapped three times, and another child was mapped four times, while still under the age of 3. Motor mapping data from the seven children included here have been reported previously (20, 31). We also identified a separate cohort of 13 children between the ages of 5 and 6 years in whom TMS language mapping was attempted. In addition to TMS, most patients underwent continuous scalp video EEG monitoring, MEG for the localization of epileptiform discharges and somatosensory and language cortices, anatomical and functional MRI, and neuropsychological testing as part of the clinical evaluation (33). Wada or CSM were not attempted in this young cohort except in one child (2.3-year-old female) who underwent subdural grid placement for localization of epileptogenic focus. The details of the demographics and diagnoses of the children in the TMS motor and language mapping groups are listed in Table 1.

**Table 1:** Demographic and clinical parameters in the motor and language mapping cohorts.

**Demographic and clinical features**	**Upper extremity mapping**	**Lower extremity mapping**	**Language mapping**
Number of patients	36	18	13
Average age ± SD (years)	1.68 ± 0.8	2.1 ± 0.5	5.6 ± 0.3
Age range	2 mo−3 y	1–3 y	5–6 y
Gender: Male/Female	19/17	11/7	8/5
Handedness: R/L/Ambi	9/8/1	8/3/1	8/3/2
Handedness: Too young/Not reported	16/2	5/1	0/0
Cortical dysplasia	8	3	2
Tuberous Sclerosis Complex type 2	8	5	1
Ischemia/Stroke	6	2	1
Infection	3	2	-
Brain tumor	4	1	7
Brain malformation	4	3	-
Other	3	2	-
Hippocampal sclerosis	–	–	1
Normal MRI	–	–	1
Lesioned Hemisphere: Left	12	3	7
Lesioned Hemisphere: Right	15	8	4
Lesioned Hemisphere: Bilateral	9	9	1
Number of AEDs (Average ± SD)	2.7 ± 1	2.9 ± 1	1.4 ± 0.5

*SD, standard deviation; R, right; L, left; Ambi, ambidextrous; AED, Antiepileptic drugs*.

### Structural MRI

Structural MR images were obtained on a 3 Tesla Siemens Verio scanner (Siemens AG, Munich, DE) or GE Signa HDxt scanner (General Electric, Milwaukee, WI) in all children utilizing sedation. In the Siemens Verio scanner, a T1-weighted 3D Stealth sequence was acquired using a 12-channel head coil (TR/TE/flip angle = 1900/2.93/9°) with slice-select inversion recovery pulses (TI = 900 ms), FOV = 512 x 512 x 176, and voxel size 0.5 × 0.5 × 1 mm. In the GE Signa HDxt scanner, a T1-weighted 3D Fast Spoiled Gradient-echo (FSPGR) sequence was acquired using an 8-channel head coil (TR/TE/flip angle = 7.95/3.56/12°), FOV = 512 × 512 × 220, and voxel size 0.5 × 0.5 × 0.8 mm. The anatomical MRI was used for neuro-navigation during TMS sessions. During the same MRI session, patients also completed other clinical MRI and fMRI sequences as part of their epilepsy evaluation.

### Transcranial Magnetic Stimulation (TMS)

Motor and language mapping were performed using an MRI-guided TMS system (NBS system 4.0; Nexstim, Inc., Atlanta, GA). The system uses a figure-of-eight coil with an outer winding of 70 mm that stimulates ~1–2 cm^2^ of the cortex beneath its central junction and had a maximum E-field of 172 Volts/meter at a distance of 25 mm from the coil surface (34, 35). The depth of stimulation is determined in each case by peeling the modeled scalp and skull until the cortical surface is visualized and ranged from 10 to 25 mm. The strength of the E-field is calculated taking into account the peeling depth, the size and shape of the individual's head, and the coil orientation parallel to the cortical columns (35) and is displayed for the chosen peeling depth. The high-resolution T1-weighted MRI of each patient was co-registered to the patient's head using anatomical landmarks and the surface matching procedure implemented in the Nexstim NBS system.

#### Motor Mapping

**Figure 1:**
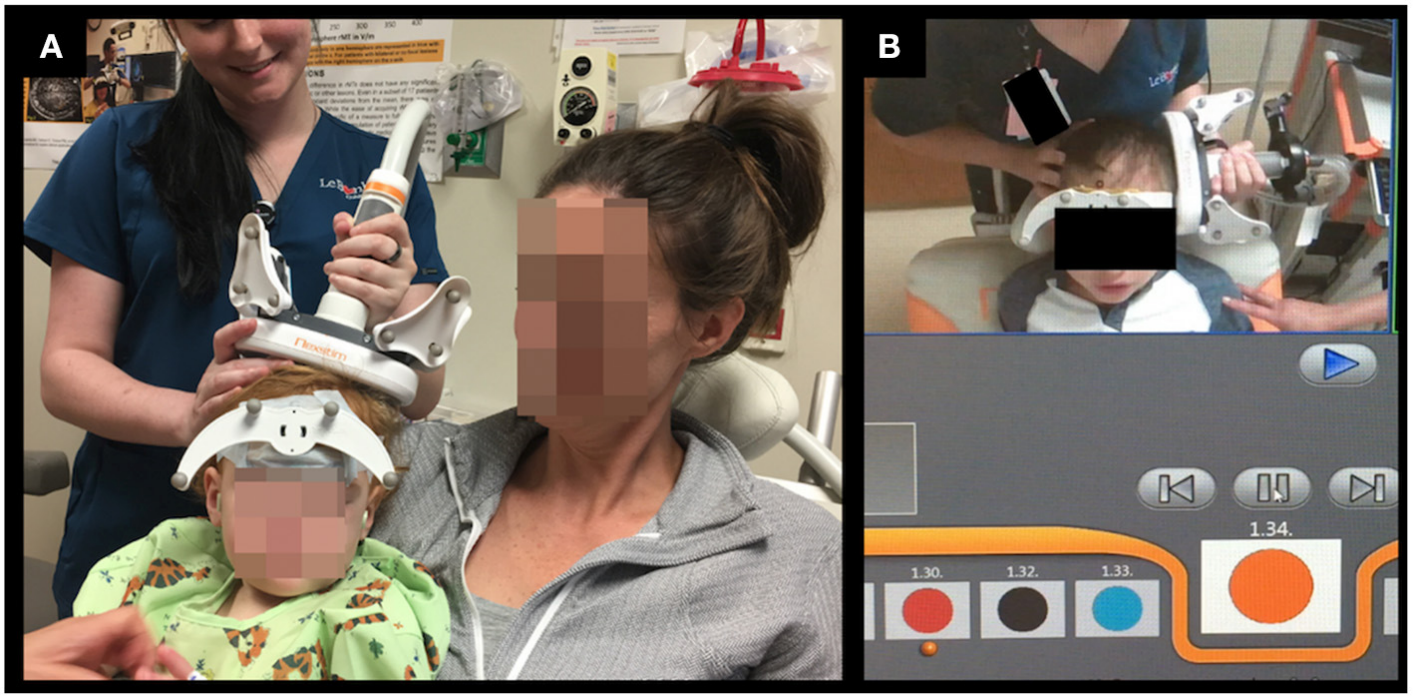
Setup for TMS motor (A) and language (B) mapping studies in young children. **(A)**: During TMS motor mapping in infants and toddlers, the child is seated on the parent's lap. **(B)**: During TMS language mapping color naming task was used in some preschool children.

TMS motor mapping was performed while the children were seated on their parent's lap (see Figure 1A for example). The children were allowed to play with toys or to watch TV during the study. The motor evoked potentials (MEPs) elicited by TMS were recorded by surface electromyography (EMG) from bilateral adductor pollicis brevis (APB), brachioradialis, and tibialis anterior (TA) muscles using disposable electrodes (Neuroline 720, Ambu Inc., Maryland, USA) and sampled at 3 kHz and band-pass filtered from 10 to 500 Hz. In each hemisphere, the mapping procedure began with application of TMS at an intensity set at 100% of the stimulator output (SO) around the middle part of the precentral gyrus (i.e., the hand knob area). The starting TMS intensity was set at 100% SO to compensate for the decreased efficacy of the standard figure-of-eight TMS coil in infants and children due to their immature motor system, brain size, and brain tissue conductivity (36, 37) as well as to reduce testing time due to the limited cooperation expected in this cohort. In children with difficulty tolerating this stimulation intensity, SO was decreased gradually until adequate tolerability was achieved. At this point, if no MEPs or CSPs were elicited, the mapping session was ended. TMS stimulation was applied as one pulse at each location with stimulation repeated as needed to cover surrounding cortex, including the precentral and postcentral sulci. The leg motor cortex was mapped by applying TMS along the paracentral lobule and posterior medial frontal gyrus. As the patients could not maintain a true baseline or the incidence of MEP was variable, motor threshold determination (resting or active) was not attempted. The TMS time-locked EMG epochs were analyzed offline to determine the presence of MEP, and, when applicable, to calculate its latency and peak-to-peak amplitude. Since the patients could not maintain relaxed muscles and had ongoing muscle contractions during TMS stimulation, we also examined the EMG recordings for any interruption of this voluntary activity following TMS, i.e., the cortical silent period (CSP) (38). CSP has proven to be a useful diagnostic biomarker in many neurological disorders including epilepsy in adults (39–42). We and others have previously shown that CSP can also be used to localize the motor cortex in individuals including young children in whom the SO required to elicit an MEP is at or near 100% (12, 20, 43).

#### Language Mapping

TMS was used to localize the language-specific cortex by employing the “virtual lesion” paradigm (44) in 13 pre-school children between the ages of 5 and 6 years. Twelve of the 13 children also completed motor mapping prior to language mapping. The color-naming task was used in eight children (see Figure 1B for example) and an object-naming task was used in five children. We have previously shown that young and developmentally delayed children who could not consistently name objects could still name colors accurately and have successfully used the color-naming task to successfully map speech and language in this cohort (32, 45). In other children, pictures of objects included in the NexSpeech module was used (46). Since children were able to name common objects or colors used in this study, we did not individualize the stimuli based on their linguistic abilities.

The participants were seated in a chair and viewed the stimuli on a monitor. Colors (or objects) were displayed for 1,000 ms with an interstimulus interval adjusted according to the individual participant's ability ranging from 3.5 to 5 s. Patients were asked to correctly name the colors or the drawings of common objects as quickly as possible. Colors/pictures erroneously named were removed from the stimulus pool, so that all stimuli presented during TMS had a corresponding correct baseline recording. The TMS SO was adjusted to deliver an E-field of 80–100 V/m at the cortical surface based on previous reports (47, 48) and our own experience in older children and adults (45, 49). Patients were continuously monitored visually and by electromyography for signs of the intracortical spread of excitation or seizures (50). Discomfort during TMS was evaluated using the visual analog scale (VAS) for pain, recorded at baseline, four times throughout mapping, and whenever a patient spontaneously expressed discomfort. The intensity was decreased if participants rated pain ≥3 on the VAS but not below an E-field of 50 V/m. The mapping was discontinued if the pain persisted, and E-field fell below 50 V/m. Stimulus presentation and TMS onset were simultaneous with no delay to include early cortical activity (51) and decrease false negative results (52). The TMS train frequency was set at 5 Hz (five pulses). For mapping language cortices, TMS was applied from the supramarginal and angular gyri and extending to the superior, middle, and inferior temporal gyri, as anteriorly as the patient could tolerate. Then stimulation of the middle and inferior frontal gyri, including the pars opercularis and pars triangularis, as well as premotor regions was attempted. Baseline performance, stimuli presented, participant's response, and cortical locations of TMS were recorded for *post-hoc* analysis. Across compliance levels, the entire language mapping procedure was completed in ~1 h. In each participant, the hemisphere to be stimulated first was decided by prioritizing clinical need and therefore, the lesional hemisphere was mapped first.

### Data Analysis and Statistical Procedure

#### Motor Mapping

**Figure 2:**
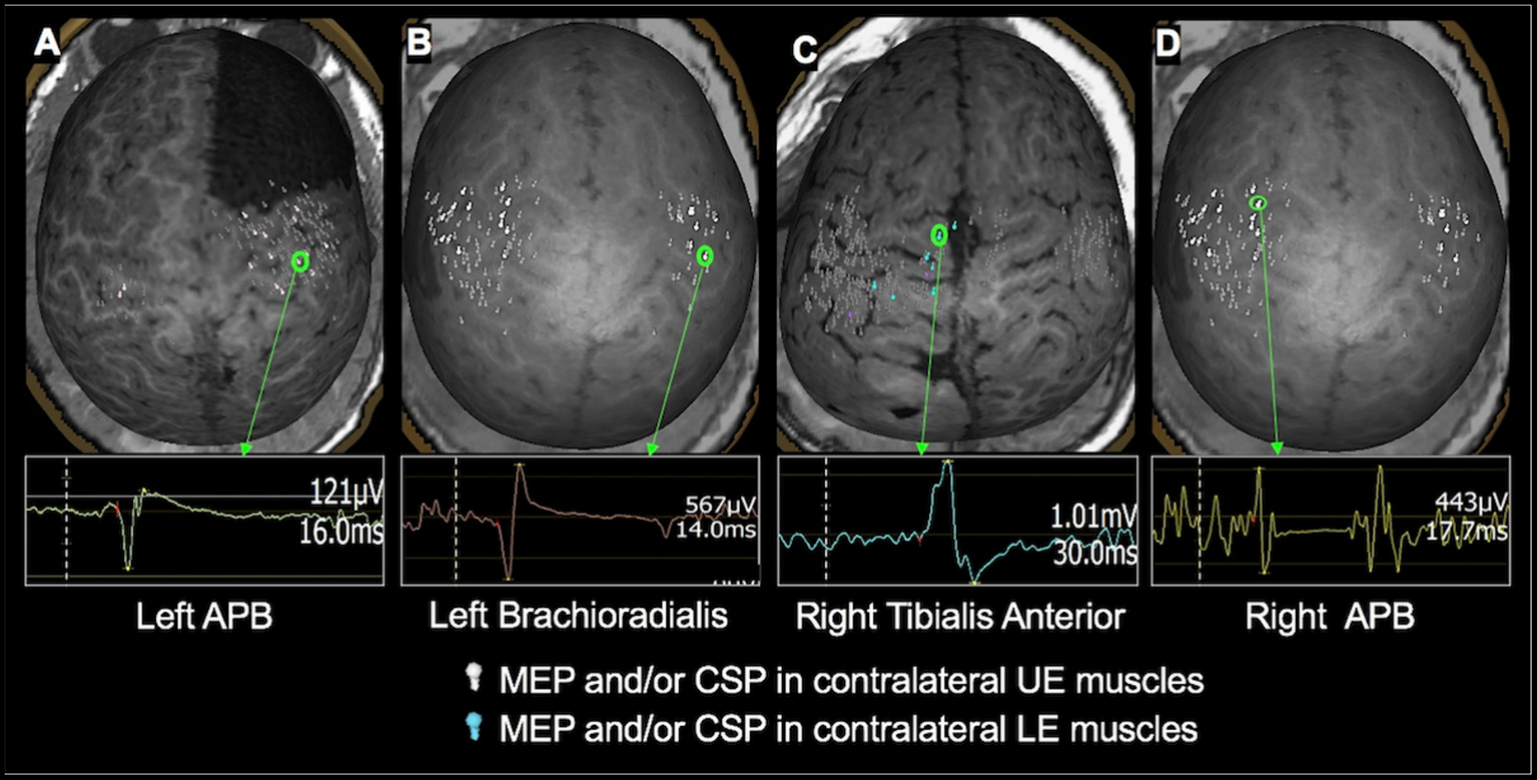
Examples of TMS-evoked MEPs and CSP in children under 3 years of age. **(A)**: A 2.4-year-old male with right frontal lobe cortical dysplasia status post resection. MEP in the left APB muscle having an amplitude of 121 μV and a latency of 16 ms was evoked when TMS was applied to the right precentral gyrus. **(B)** An 18-month-old female with history of left hemisphere perinatal stroke involving left temporal lobe and subsequent infantile spasms demonstrating MEP evoked in the left brachioradialis muscle with an amplitude of 567 μV and a latency of 14 ms following TMS applied to the right precentral gyrus. **(C)** 1.7-year-old female with TSC- 2 demonstrating the MEP elicited in the right tibialis anterior muscle having an amplitude of 1.01 mV and a latency of 30 ms when TMS was applied to the left medial frontal lobe. **(D)** An 18-month-old female with history of left hemisphere perinatal stroke involving left temporal lobe and subsequent infantile spasms demonstrating CSP evoked in the right APB muscle following TMS applied to the left precentral gyrus. The left hemisphere is on the left side of the image.

Motor mapping recordings of the EMG were reviewed, and cortical locations where MEPs were observed on the EMG were identified by comparing it with the patient's baseline EMG data, as well as with previously established criteria for identification of cortical motor cortex responses (50). For each MEP, peak-to-peak amplitude and corticomotor latency (time from TMS stimulation to MEP onset) were initially estimated using the automated algorithm included in Nexstim software and verified by visual inspection. The MEP amplitude was used as a threshold to determine whether TMS elicited a response or not. Since the EMG electrode positions were variable between children and the baseline EMG also varied greatly, from being quiet (asleep or paresis) to vigorously active (holding a toy, being agitated, etc.), the MEP amplitudes were not further analyzed. When present, the onset and offset of CSP were calculated by visual inspection. When CSP was observed following an MEP, the MEP onset was also considered to be the CSP onset. When isolated CSP was observed, its onset was determined to be the point when EMG became quiet, and its offset as when the EMG activity returned to baseline pre-TMS levels. Similar to MEPs, the presence of CSP was used to localize response to TMS, and no further analysis was carried out. Examples of TMS-elicited MEPs and CSPs in APB, brachioradialis, and TA muscles are shown in Figure 2. Cortical locations of identified MEPs and CSPs were then marked on the patient's MRI.

#### Language Mapping

Videos of patient performance during TMS language mapping were reviewed and potential speech and language errors were compared with the corresponding baseline response for the same item. Observed errors were independently categorized using the recommended criteria (9, 53) by two authors blinded to the site of stimulation. Any disagreement in categorization was resolved by consensus. The performance was coded as follows: *speech arrest errors*: when TMS resulted in an inability to produce any response; *semantic errors*: when a semantically related or associated word is substituted for the target word; and *performance errors*: form-based distortions, slurring, stuttering, imprecise articulation, or delayed response when compared to baseline recordings of the patient naming the same color or object. Speech errors attributed to discomfort or distraction were excluded from analysis. Further, speech errors resulting from the stimulation of primary mouth and laryngeal motor cortices or lips, jaw, and tongue muscles were also removed. The cortical location, type of error, and TMS intensity (%SO and E-field) for each TMS train were recorded. Similar to language mapping by fMRI (54, 55), we have previously shown that TMS-induced speech and language errors are also amenable to calculating a laterality index (LI) (45, 49). The LI was calculated as E_left_-E_right_/E_left_+E_right_, where E_left_ was the total number of speech and language errors in the left hemisphere, and E_right_ was the total number of speech and language errors in the right hemisphere. We also calculated LI by weighing the speech arrests (3x) and semantic errors (2x) more than the performance errors. Similar to LI thresholds used in fMRI (54, 55), TMS LI values 1.0–0.1 indicated left hemisphere dominance (HD), −1.0 to −0.1 indicated right HD, and values between −0.1 and +0.1 were considered a balanced bilateral representation of language.

## Results

### Motor Mapping

#### Patients

The motor mapping cohort (*n* = 36 children, 47 total maps) consisted of 19 males and 17 females with a mean age (± SD) of 1.68 (± 0.8) years. The youngest child studied was a 2-month-old female, and 13 children were aged younger than 1 year. Nine children were reported to be right-handed, eight were left-handed, one was ambidextrous, two were not reported, and 16 were indeterminate due to their young age. Each child's handedness was determined *via* clinical evaluation by their attending neurologist. Patients were evaluated for refractory epilepsy (*n* = 32) or a brain tumor (*n* = 4). The most common etiologies for seizures were tuberous sclerosis complex type 2 (TSC-2, 22%), cortical dysplasia (22%), and perinatal stroke (17%). All children had an identifiable lesion on the MRI, with the lesion found in the left hemisphere in 33%, in the right hemisphere in 42%, and bilateral in 25% of the children. Thirty-two children were on AEDs (average 2.7 ± 1; range 1–5). The most common AEDs prescribed were levetiracetam (51% of children), clobazam (30%), oxcarbazepine (30%), and lacosamide (26%). The details of the demographic, clinical, and AED information of the motor mapping cohort are listed in Table 1.

#### Mapping Success

Upper extremity (UE) primary motor cortex localization was attempted in both hemispheres in 40 sessions, in only the hemisphere of clinical interest in four, and in the only intact hemisphere in three. Six children were mapped twice (range 2–13 months apart), one child mapped three times (at ages 0.4, 1, and 2 y), and another child mapped four times (at ages 0.5, 0.6, 0.9, and 2.4 y). Motor mapping was considered successful if MEP or CSP was observed even for one stimulation. Three children had only one clear response in each hemisphere, but most often, five or more clear responses were observed (average number of responses = 23; 14% of stimulations). On average, 167.2 stimulations were delivered per session with a range of 26–535 single pulses. Using these criteria, the motor representation for APB and brachioradialis muscles were successfully localized in at least one hemisphere in 44 sessions (33 children) and in both hemispheres in 33 sessions (27 children). In two children, a 5-month-old with cortical dysplasia and a 7-month-old with a tumor, TMS did not elicit any MEPs or CSPs in APB or brachioradialis muscles despite extensive stimulation of both hemispheres at 100% SO. In the 5-month-old infant, the repeat motor mapping at 11 months of age was successful. Finally, the hand/forearm motor cortex could not be identified in one 10-month-old infant with non-lesional refractory epilepsy who could not tolerate increasing TMS intensity > 70% SO.

The primary leg motor cortex localization was attempted in both hemispheres in 18 children (11 males, seven females; average age ± SD: 2.1 ± 0.5 y). Two children were mapped twice. The youngest child studied was a 1-year-old male, and nine children were younger than 2 years. The motor representation of TA was successfully localized in both hemispheres in eight children, with only one hemisphere successfully mapped in seven children. In five children (four males; average age ± SD: 2.0 ± 0.8 y), despite extensive stimulation of both hemispheres at 100% SO, TMS did not elicit any MEPs or CSPs in the lower extremity muscles.

#### Safety of TMS

All mapping sessions were completed under nursing supervision. We were able to accurately apply TMS in all children as they were seated in their parent's lap. In all children, except for the one child noted above, the loud TMS clicks and the sensation of tapping at an intensity of 100% SO was well-tolerated without any serious adverse effects. We did not use earplugs in an attempt to minimize exposure to TMS clicks as the children could not keep them in place. The entire motor mapping session, including placing EMG electrodes, registering to the MRI, and surveying brain areas, was completed most often in 30–45 min. Five children (four males, age range 0.4–2.9 y) had seizures during the motor mapping procedure, and five (three males, age range 1.7–2.6 y) had seizures after the mapping procedure was completed, while being transferred to the wheelchair or transported to their inpatient room. In all children, the seizure semiology and duration during/following TMS were consistent with their clinical seizures observed at home and/or in the epilepsy monitoring unit, and all had a history of refractory epilepsy with frequent seizures. The seizures were either focal motor involving face and/or extremity twitching (*n* = 5) or generalized atonic seizures characterized by drop attacks (*n* = 5). In all children, seizures lasted <1 min (range 5 s−50 s) and did not require administration of rescue medication. In children who had seizures during mapping, the procedure was successfully completed, and meaningful data were derived.

#### TMS Parameters

Over 47 motor mapping sessions, an average of 167 ± 89 stimulations (range 26–532) with an intensity of 98 ± 8% SO was applied. The equivalent E-field in the UE motor cortex was 243 ± 97 V/m and in the leg motor cortex was 209 ± 73 V/m, both measured at a peeling depth of 17 ± 3 mm. There was no significant difference in the TMS E-field between the left and right hemispheres. The corticomotor latency from motor cortex to APB was 17.8 ± 3.1 ms; for brachioradialis, 16.6 ± 4.6 ms; for TA, 26 ± 4.5 ms. When isolated CSPs were observed, the latency for UE muscles was 34.1 ± 4.7 ms and for LE muscles 37.7 ± 6.7 ms. The details of the TMS parameters for upper and lower extremity mapping are listed in Table 2.

**Table 2:** TMS parameters in the motor and language mapping cohorts.

**TMS parameters**	**Upper extremity mapping**	**Lower extremity mapping**	**Language mapping**
Number of sessions—attempted	47	20	13
Number of sessions—successful	44	15	12
Number of stimulations—single pulse	167 ± 89		191 ± 97
Number of stimulations−5 Hz	n/a		130 ± 52
TMS intensity—% MO	98 ± 8	98.5 ± 7	34 ± 3
TMS intensity—E field (V/m)	243 ± 97	209 ± 73	91 ± 14
Corticomotor latency—APB (ms)	17.8 ± 3.1	n/a	n/a
Corticomotor latency—Brachioradialis (ms)	16.6 ± 4.6	n/a	n/a
Corticomotor latency—TA (ms)	n/a	26 ± 4.5	n/a
Motor mapping: Normal localization	30	15	12
Motor mapping: Developmental variant	6	–	0
Motor mapping: Cortical reorganization	8	–	0
Language mapping task: Colors	n/a		8
Language mapping task: Objects	n/a		5
Number of speech arrests (Average ± SD)	n/a		4 ± 5
Number of semantic errors (Average ± SD)	n/a		2 ± 2
Number of performance errors (Average ± SD)	n/a		10 ± 7
LH dominance	n/a		-
RH dominance	n/a		5
Bilateral dominance	n/a		1
Dominance not determined	n/a		6
Adverse effects - pain at site of stimulation	1		6
Adverse effects - seizures	10		0

*SO, stimulator output; E-field, Electric field; APB, Adductor pollicis brevis; TA, Tibialis anterior; SD, standard deviation; LH, left hemisphere; RH, right hemisphere; n/a, not applicable*.

#### Localization of Motor Cortex

**Figure 3:**
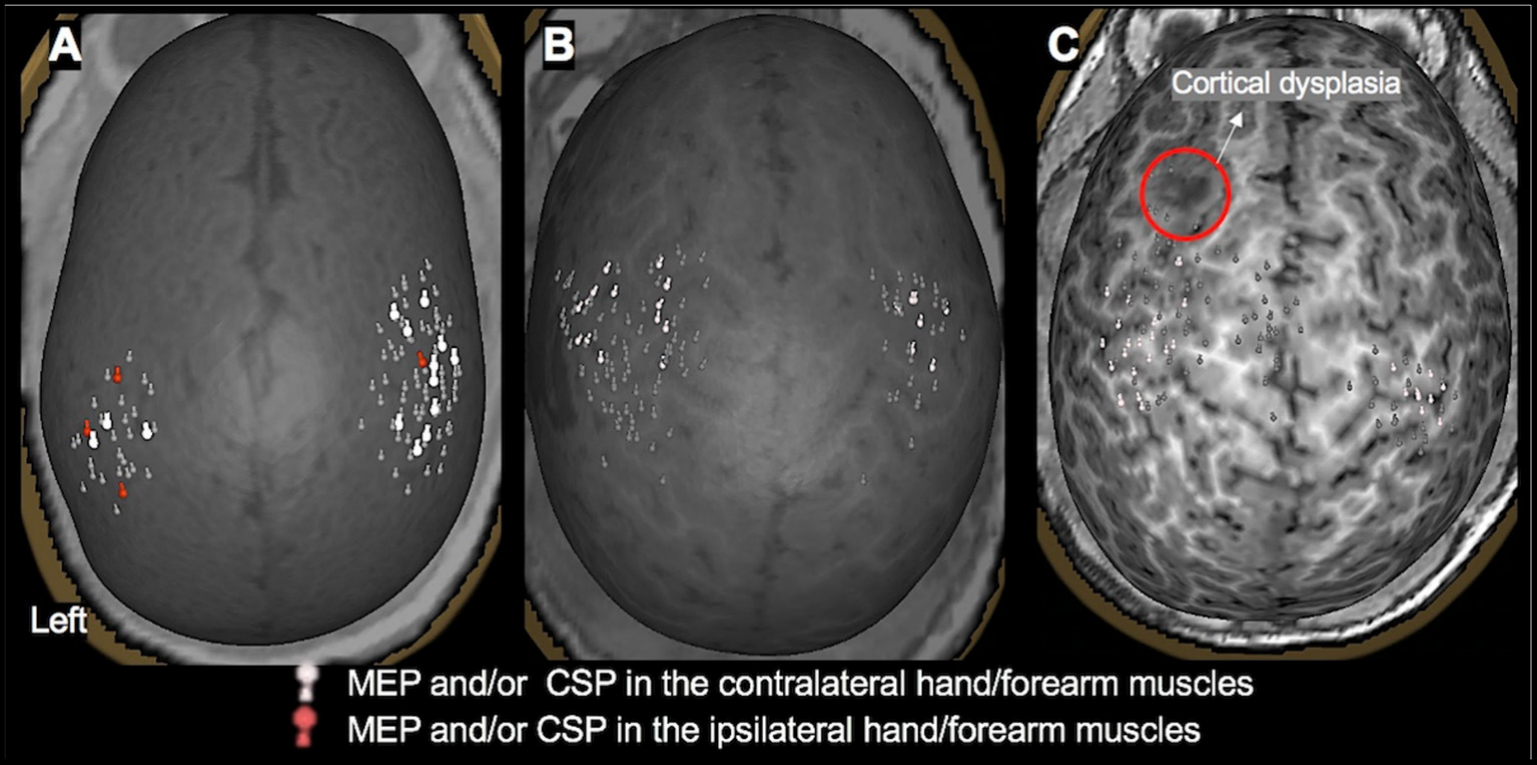
TMS motor mapping demonstrating normal motor development in children under 3 years of age. **(A)**: A 3-month-old male with dysplasia along the inferior frontal sulcus involving the inferior aspect of the right precentral gyrus, inferior gyrus, frontal gyrus, and middle frontal gyrus and history of infantile spasms. The motor cortices were localized along the precentral gyrus with MEPs elicited in contralateral and ipsilateral hand muscles, representing a normal developmental variant. **(B)**: An 18-month-old female with history of left hemisphere perinatal stroke involving left temporal lobe and subsequent infantile spasms demonstrating a normal motor map. **(C)**: A 2.4-year-old female with history of left frontal lobe focal cortical dysplasia, type IIb. Motor representation was localized posterior to the dysplasia.

**Figure 4:**
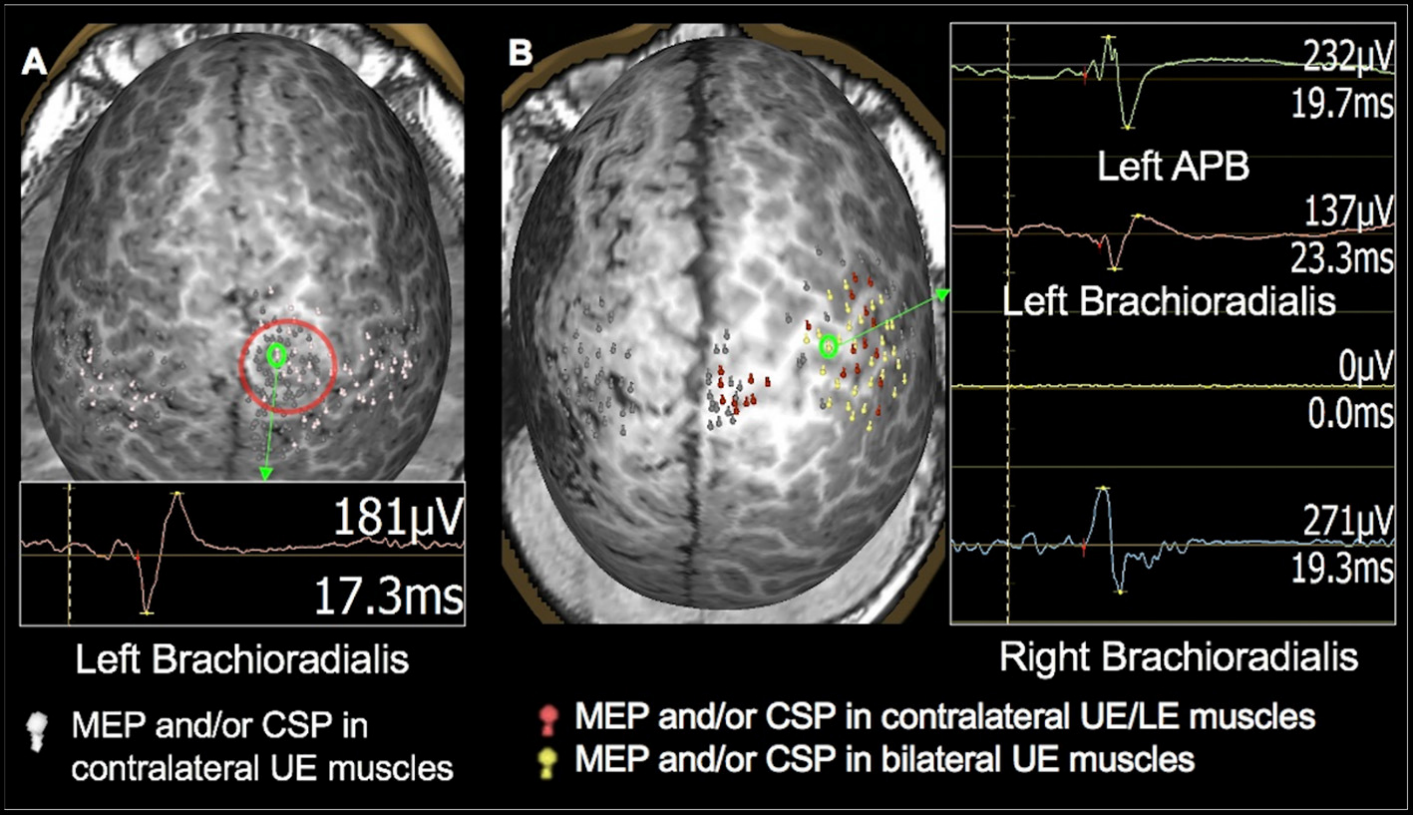
TMS motor mapping demonstrating cortical reorganization in children under 3 years of age. **(A)**: TMS motor mapping in a 1.7-year-old male with history of refractory seizures involving left-sided tonic flexion with a cortical dysplasia in the medial frontal side of the right frontal lobe on the pre- and post-central gyrus. TMS localized left hand and forearm representation to the precentral gyrus. Additionally, MEPs in the hand and forearm muscles were elicited while stimulating the area of cortical dysplasia. No MEPs were elicited in the left lower extremity even at 100% of stimulator output. The child underwent surgical resection of the lesion. At 9 months follow up, he was seizure-free with intact left-hand function and mild left leg monoparesis. **(B)**: A 2-year-old male with history of left hemisphere perinatal stroke and right hemiparesis presenting with refractory epilepsy. TMS motor mapping demonstrated no motor representation for right upper extremity in the left hemisphere. Instead, both left and right upper extremities were represented around the precentral gyrus in the right hemisphere. The left hemisphere is on the left side of the image.

In the 33 children in whom motor mapping was successful, the UE primary motor cortex was localized to the central part of the precentral gyrus around the hand knob area (See Figure 3 for examples). In five of these children (one with TSC-2, one with infection, and three with cortical dysplasia), stimulation of both hemispheres resulted in occasional MEPs in both UE muscles, representing intact uncrossed pyramidal neurons, a normal variant in this age group (see Figure 3A). In four out of six children with perinatal stroke, an interhemispheric pattern of motor reorganization was demonstrated with bilateral UE motor representation in the intact hemisphere (see Figure 4B). In two out of eight children with cortical dysplasia, an intrahemispheric pattern of motor reorganization was demonstrated with the UE representation displaced toward the lower extremity motor cortex (see Figure 4A) or the premotor cortex. In 15 children in whom LE mapping was successful, the primary leg motor cortex was localized to the paracentral lobule in the medial frontal gyrus in one or both hemispheres.

#### Comparison of TMS-Derived Motor Maps Against Other Mapping Modalities

**Figure 5:**
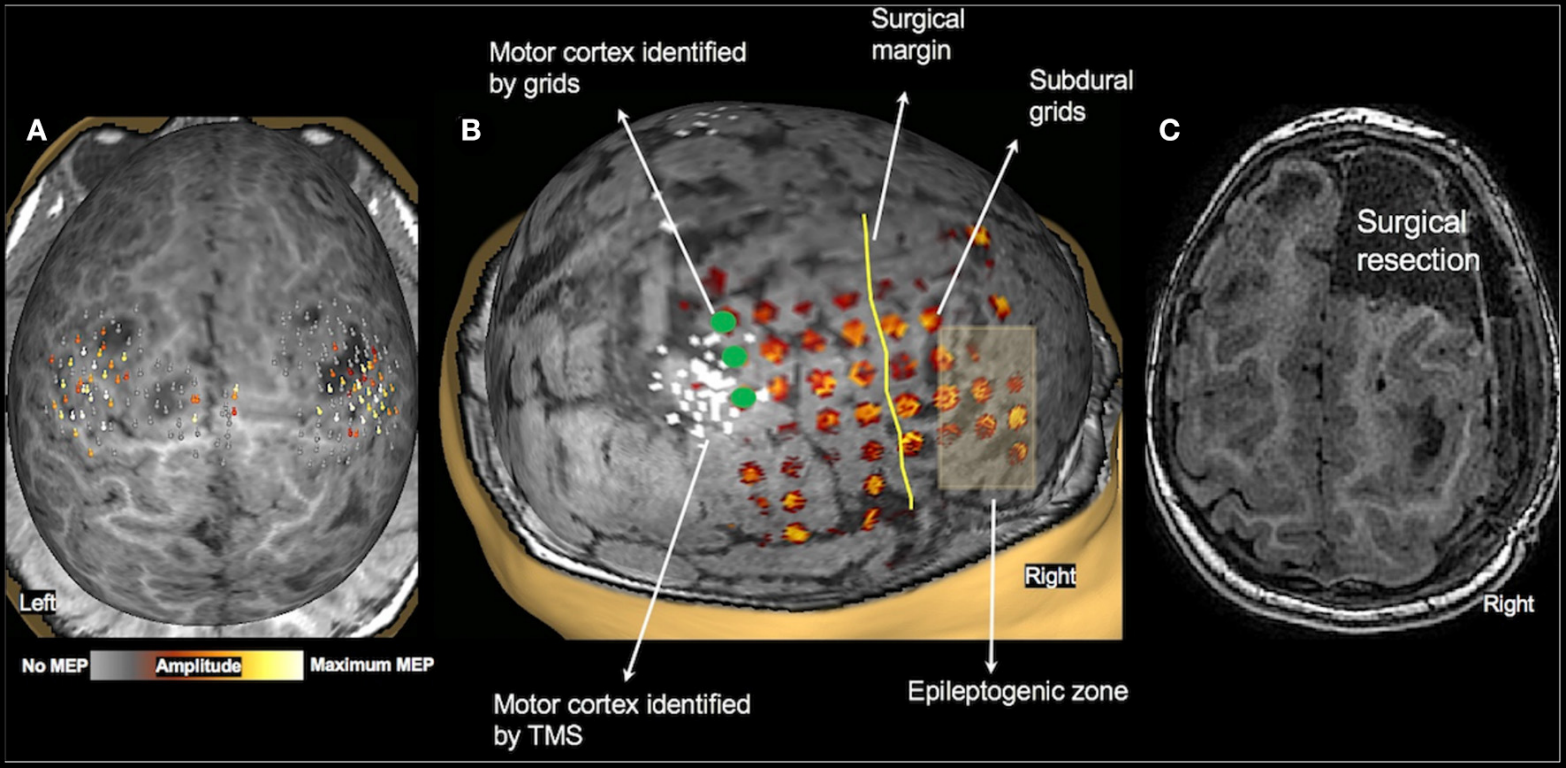
Validation of TMS motor mapping by CSM. Presurgical TMS-derived motor mapping in a 2.3-year-old female with tuberous sclerosis complex type 2. **(A)**: TMS localized motor cortex in the right hemisphere in the precentral gyrus in the vicinity of a tuber. **(B)**: The child underwent subdural grid placement, and the epileptogenic focus was localized to be anterior to the motor cortex. **(C)**: The child underwent right anterior frontal lobectomy including the epileptogenic focus. Post-operatively, the child moves all extremities equally with normal bulk and strength and uses either hand to reach for an object.

Of the 36 patients who underwent TMS motor mapping, 20 children underwent somatosensory mapping with MEG, all under sedation. MEG was successful in only five children (four bilateral, and one in one hemisphere only). Twenty-five children underwent fMRI during passive hand movement, also under sedation. The sensorimotor cortex was successfully mapped in 20 children (18 bilateral, and two in one hemisphere only). These findings are consistent with our previous report in a smaller cohort of children under 3 years of age (20). When compared to TMS, which assesses motor cortices directly, MEG and fMRI under sedation primarily mapped the somatosensory cortex. Although the primary motor and sensory cortices are closely linked and findings of one modality can, to some extent, be generalized to the other, the comparison is not a direct one. Therefore, we did not use fMRI or MEG somatosensory maps as controls for TMS motor results. One child (2.2 y/o female with TSC-2, see Figure 5) underwent subdural grid placement to confirm the epileptogenic focus. In this child, the CSM- and TMS-localized motor cortices showed excellent overlap (Figure 5B). None of the other children underwent invasive mapping. We therefore attempt to demonstrate the utility and accuracy of TMS presurgical motor mapping in this cohort through its use in surgical planning and the post-operative results.

#### Surgical Intervention

Of the children who underwent motor mapping only, many did not proceed to surgery. TMS mapping in these children provided the parents and physicians with information regarding the location of the child's motor cortices and also provided a baseline status of motor development. In children who returned for repeat motor mappings, TMS results were used to track motor function and development over time. Motor mapping was also used to assess the risks and benefits of surgical treatment in 11 children who had lesions in the vicinity of motor cortex. They included 2 children with brain tumor, 6 children with cortical dysplasia, 2 children with TSC-2, and one child with perinatal stroke. In all children who underwent surgery, the MRI with the TMS locations marked was transferred to the surgical navigation system, and the proximity of the anatomical lesion and/or the epileptiform focus to the primary motor cortex identified by TMS was estimated. The children were evaluated clinically at follow up and 7 children were found to have good motor function with no new motor deficits. The child with perinatal stroke, in whom motor representation in the lesioned hemisphere was shown to be reorganized to the intact hemisphere, demonstrated an improvement in preexisting hemiplegia following surgery. One child had slightly decreased movement in the contralateral UE with good muscle bulk, strength, and tone. Two children were found to have mild monoparesis at follow up; in one, the resected cortical dysplasia was within the primary leg motor cortex (Figure 4A), and in the other, the frontal lobectomy extended up to the precentral sulcus (Figure 2A).

### Language Mapping

#### Patients

The TMS language mapping cohort consisted of eight males and five females with a mean age (± SD) of 5.6 (± 0.3) years, with all children being between 5 and 6 years of age. Eight patients were right-handed, three left-handed, and two ambidextrous. The patients were being evaluated for tumor (*n* = 7) or refractory epilepsy (*n* = 6). The causes of refractory seizures were cortical dysplasia (*n* = 2), tuberous sclerosis complex type 2 (*n* = 1), perinatal stroke (*n* = 1), and hippocampal sclerosis (*n* = 1). The lesion was in the left hemisphere in seven children, in the right hemisphere in four children, bilateral in one child, and no detectable lesion on MRI in one child. Ten children were on AEDs (average 1.4 ± 0.5; range 1–2). The most common AED prescribed was levetiracetam (50%). The details of demographic, clinical, and AED information of the language mapping cohort is listed in Table 1.

#### Mapping Success

The temporal and frontal lobes in both hemispheres were successfully mapped in six of 13 children. In five of the remaining children, bilateral temporal lobes were evaluated, but stimulation of frontal lobe language areas could not be tolerated. In one child, only the left hemisphere frontal lobe around the tumor was mapped. One child could not tolerate TMS stimulation and language mapping was discontinued. Twelve children also successfully completed motor mapping of the upper and lower extremities.

#### Safety of TMS

We were able to map at least part of the language areas in the two hemispheres in 92% of this cohort, with comprehensive language maps derived in 50% of children. The most common complaint was pain during stimulation. The TMS intensity was reduced when the pain reported on VAS was ≥3. Throughout the mapping procedure, we ensured that the pain score was below 3. No child had a seizure during or after completion of the TMS language study.

#### TMS Parameters

Eight children were mapped using the color-naming task and five using the object-naming task. The TMS parameters of rate and intensity used in this study were within the guidelines for safety (50). An average of 130 (± 52) trains of 5 Hz stimulation were applied. The TMS intensity was 34 ± 3 % SO (range 25–40% SO), equivalent to an E-field of 91 ± 14 V/m (range 58–107 V/m), measured at a peeling depth of 17 ± 3 mm. The hemisphere with the lesion was stimulated first (LH: 9; RH: 3). Mapping was considered successful if at least one convincing speech error was noted; however, the lowest number of errors noted in any child was six (both hemispheres included). On average, 13% of stimulations resulted in speech errors. Performance error (10 ± 7) was the most common type of speech error noted, followed by speech arrest (4 ± 5), and then semantic errors (2 ± 2). The average total number of errors in the left and right hemispheres were similar (LH: 8.1 ± 6.7; RH: 8.2 ± 7.8). The TMS intensities recorded were not significantly different for the three error types, indicating that the type of speech errors elicited by TMS were independent of TMS intensity. The details of the language mapping TMS parameters are listed in Table 2.

#### Localization of Language Cortices

**Figure 6:**
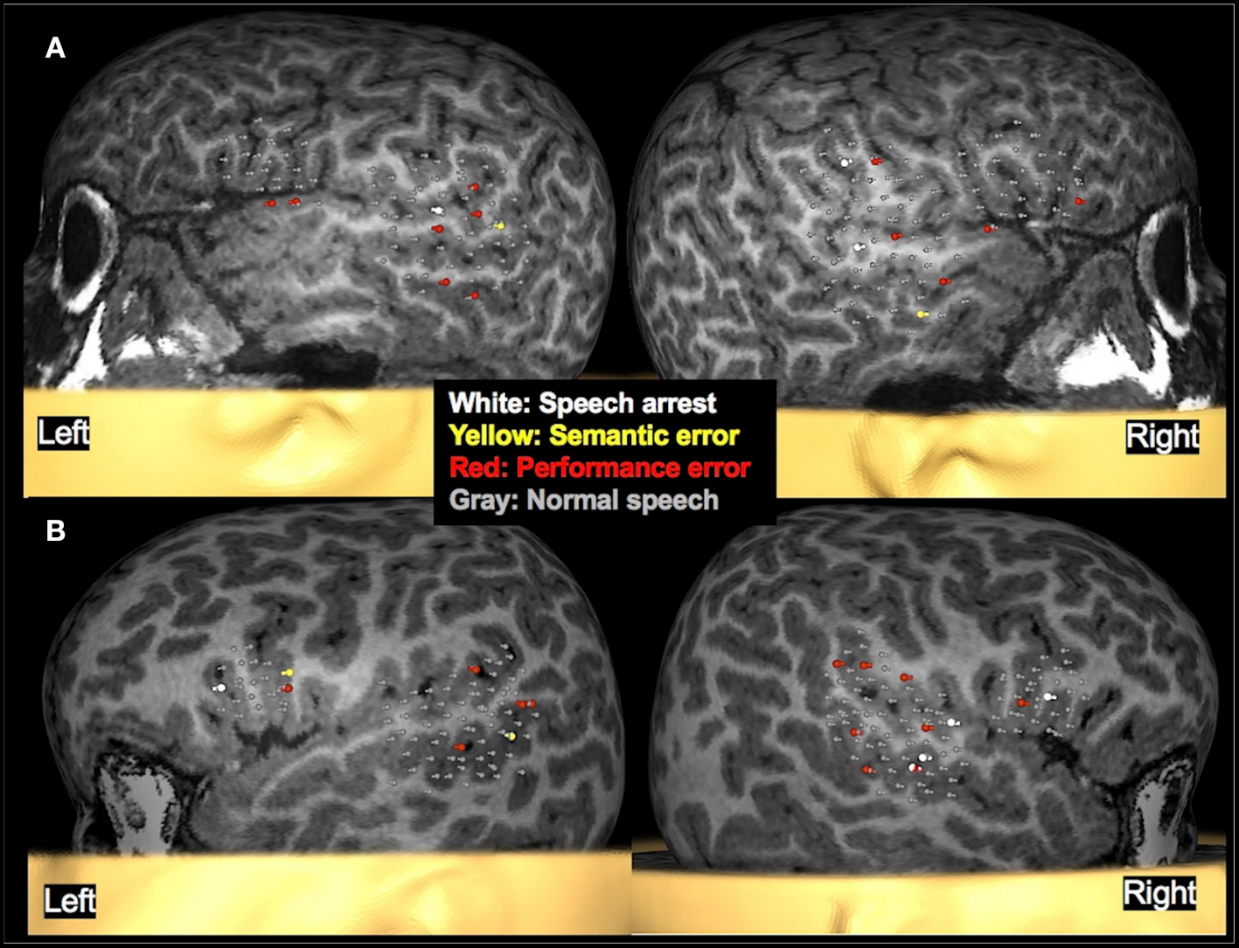
Examples of language mapping with TMS. Speech errors in the form of speech arrest, semantic errors, and performance errors were elicited in both hemispheres. **(A)**: Language mapping with TMS in a 5.6-year-old female with refractory cryptogenic focal epilepsy and asymptomatic cervical and thoracic syringohydromyelia. Her brain MRI was normal. TMS language mapping was completed using a color naming task and showed bilateral dominance for expressive language. **(B)**: Language mapping with TMS in a 5.6-year-old male with right parietal cortical dysplasia that was in the inferior parietal lobule, predominantly superior to the marginal gyrus. TMS language mapping was completed using an object naming task and indicated a right hemisphere dominance for expressive language.

**Figure 7:**
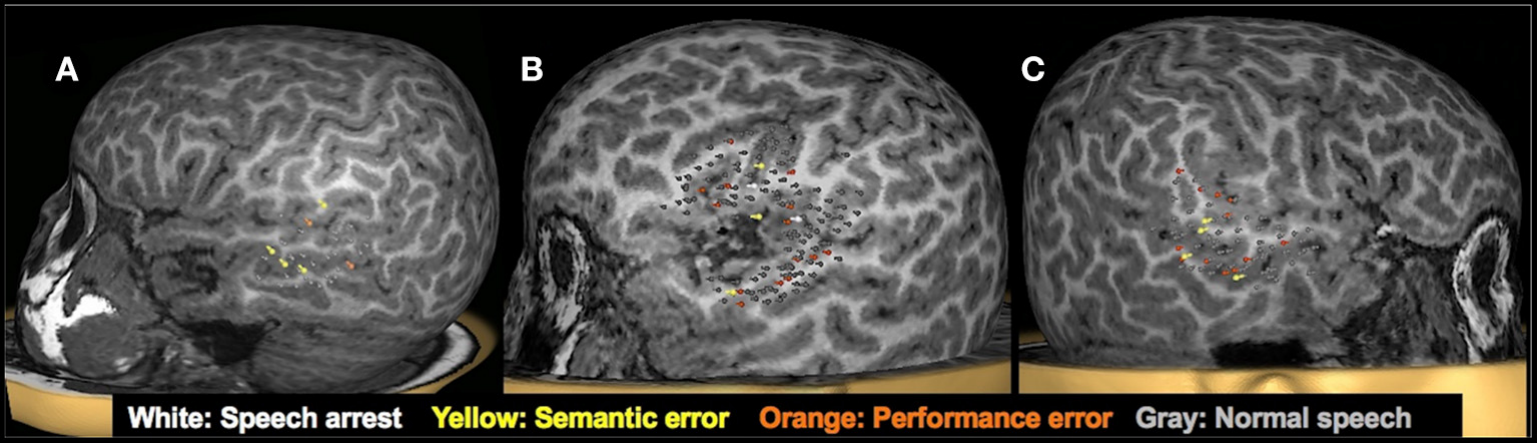
Clinical utility of presurgical TMS-derived language mapping in preschool children with brain tumors. **(A)**: Left hemisphere temporal lobe language mapping in a 5.3-year-old female with recurrent pilomyxoid astrocytoma. She underwent a left temporal microsurgical subtotal (70%) tumor resection. Post-operatively, she had no speech deficits. **(B)**: TMS language map from a 5-year-old male with recurrent left sylvian anaplastic ependymoma. Critical language areas were found around the margin of the tumor. The tumor was resected in full without any postoperative language deficits. **(C)** Right hemisphere temporal lobe language mapping in a 5.9-year-old female with a lesion in the right temporal lobe. Critical language areas were identified in bilateral temporal lobes. She underwent a resection of the right anterior temporal lobe, right amygdala, and hippocampus. The pathology classified the specimen as grade I ganglioglioma and focal cortical dysplasia type IIIb. Post-operatively, she had no speech deficits.

TMS-elicited speech disruptions were noted following stimulation of the middle and posterior parts of the superior and middle temporal gyri and the supramarginal gyrus in both hemispheres (see Figures 6, 7 for examples). Critical language areas identified in the frontal lobe included the ventral premotor cortex, pars opercularis, and pars triangularis (see Figures 6, 7). Across both hemispheres, language areas were primarily localized in the superior temporal lobe (83% of patients), the middle temporal gyrus (68% of patients), and the supramarginal gyrus (64% of patients). Language representation in the inferior frontal gyrus was identified in 94% of children in whom the frontal lobe was stimulated. Based on the localization of language cortices in the frontal and temporal lobes in the two hemispheres by TMS, the LI was estimated in 11 children. Two were deemed LH dominant, seven RH dominant, and two bilaterally dominant for language.

#### Comparison of TMS Derived Language Maps Against Other Mapping Modalities

Of the 13 patients who had TMS language mapping, 10 underwent MEG receptive language mapping under sedation, of which only three were successful. Of the eight children who underwent fMRI during passive listening under sedation, language cortices in the temporal lobes were successfully localized in only three patients. Because the sedation required for MEG (27) and fMRI (56) in this age group often precludes successful and reliable mapping, and even when successful, mapping primarily consists of receptive language, and the comparison against expressive language maps derived by TMS is not a viable option. None of the children in this group underwent invasive mapping. However, we feel that the utility and accuracy of TMS presurgical mapping in this cohort is best shown by its use in surgical planning and the post-operative results, presented below.

#### Surgical Intervention

Of the 13 children who underwent speech mapping with TMS, seven children underwent surgery to remove the tumor (*n* = 6) or tuber (*n* = 1). Due to the availability of TMS mapping, none underwent CSM. One child underwent placement of VNS, and the remaining five did not undergo surgery. Of those five, two declined surgery, two were experiencing adequate seizure control with medication management at that time, and one was not a surgical candidate due to non-localizable seizures. TMS mapping allowed neurosurgeons to demonstrate patient-specific functional areas and the proposed surgical approaches to patients' families, allowing both parties to accurately weigh the risks and benefits of proceeding with surgery at that time. Finally, for all children who underwent surgery, the TMS results provided a presurgical baseline of expressive language function which was used for comparison with post-operative language. In these children, the MRI with the TMS locations marked was transferred to the surgical navigation system, and the proximity of the anatomical lesion and/or the epileptiform focus to the language cortices identified by TMS was estimated. Post-operatively, the children were evaluated clinically, and none of the children were found to have speech or language deficits.

## Discussion

In this study, we demonstrate, successful localization of motor, speech, and language cortices in young children with refractory epilepsy or brain tumor using TMS. All children were tested in the awake state. Motor cortices were successfully mapped in 90% of children under 3 years of age, with TMS eliciting reliable MEPs and/or CSPs. In this young cohort, we were able to demonstrate normal developmental patterns as well as lesion-dependent cortical reorganization. In pre-school children aged between 5 and 6 years, language areas in the temporal lobes were localized in 92%, while language areas in the frontal lobes were successfully identified in 54%. To the best of our knowledge, this is the largest study reporting mapping of motor cortices in toddlers and language cortices in pre-school children using TMS. The successful TMS in these patients was in part due to the use of MRI-guided TMS and real-time localization of coil position and its orientation with respect to the cortical surface. As shown previously, when directly compared with TMS delivered without MRI guidance, navigated TMS leads to more accurate targeting of cortical areas which in turn results in more significant physiological and behavioral effects in both diagnostic and therapeutic TMS paradigms (57). The use of real-time visualization of the location and orientation of the coil along with the modeled E-field further facilitated TMS coil positioning (35).

Successful localization of motor and language cortices was helpful in optimizing risk-benefit evaluation in this population. For instance, TMS findings of bilateral language representation in the pre-school children and demonstration of the absence of motor function in the vicinity of lesions (cortical dysplasia/tumor/epileptogenic focus), respectively, increased confidence in recommending surgery. More importantly, TMS findings facilitated surgical planning aimed at preserving motor and language functions. If data from TMS were not available, the patients would have had CSM with intracranial electrodes. Due to the availability of TMS, the risks associated with placement of intracranial electrodes were avoided and the children proceeded directly to surgery. Post-operatively, motor function was preserved in most children with only two children having mild, predicted weakness. Language was intact in all seven patients who had surgery for lesions near the language cortices. The data presented here provide preliminary evidence on the utility of presurgical TMS in preserving function and improving outcomes in toddlers and pre-school children with epilepsy or a brain tumor. Our findings add to the emerging evidence on the effectiveness of TMS alone, or in combination with functional mapping methods, in predicting postsurgical outcome in adults and adolescents with epilepsy and brain tumor (8, 11, 14, 47, 58–61). Finally, TMS mapping also provided a baseline to evaluate post-surgical changes in motor, speech, and language function.

### Motor Mapping

While TMS motor mapping studies in healthy children as young as 0.2 years have demonstrated that reproducible MEP can be elicited in young children (62, 63), we demonstrate here that similar mapping is feasible in children of this age group with neurological disease. As demonstrated here, it is possible to localize primary hand and leg motor cortices and measure corticomotor latencies by eliciting MEPs and CSPs in hand and leg muscles. In this motor mapping cohort of children under 3 years of age, MEPs were elicited using maximum intensity of TMS, indicating increased activation thresholds in these children. This observation is consistent with previous studies in healthy young children, which have shown that the motor thresholds are high in the first few years of life (64) and remain high up to 10 years of age (65) due to the immature nature of motor system. Additionally, the relatively smaller head sizes and the different conductivity of brain tissues in young children influence the maximum E-field and decay over distance from the coil (36, 37). Another factor that can result in higher stimulation thresholds in this cohort is the presence of AEDs. Here, patients were on an average of 2.7 AEDs (up to 5 AEDs), ranging from sodium channel blockers or stabilizers, γ-aminobutyric acid (GABA) agonists, GABA analogs, and presynaptic calcium channel inhibitors.

One method through which we overcame the drawback of high thresholds to elicit MEP in this cohort, was the use of CSP to localize motor cortex (38, 66). The duration of the CSP is compatible with a long-lasting period of inhibition mediated by GABA receptors (67). The GABAergic interneurons mediating CSPs have lower thresholds than the pyramidal neurons that elicit MEPs (68, 69). Consistent with reports from our group (20, 31) and others (12, 70), in cases where the TMS intensity of ≥100% of SO is required to elicit MEPs, CSPs are more easily elicited. Using CSP, we were able to localize the motor cortex as well as assess the degree of inhibitory activity in the motor cortex.

The motor cortex localized by TMS has been validated against CSM-derived motor mapping in persons with a brain tumor (6, 71–73) and epilepsy (12). The mean distance between CSM- and TMS-motor locations were between 2 and 11 mm for hand muscles. Only one study has compared TMS and CSM results in children (*n* = 8; age range 9–17 years) (12). In one child in our cohort, who had subdural grid placement, we found the motor cortex identified by TMS and CSM had excellent agreement (Figure 5B). This finding provides preliminary evidence that TMS motor mapping is valid in young children and shows promise for use in children who cannot undergo invasive mapping. In our study, TMS-derived motor mapping informed in surgical planning in 11 children in whom the epileptogenic focus/tumor was in close proximity to the motor cortex. In the majority of these children, MEG and fMRI were unsuccessful and even when successful, these modalities provided information on the somatosensory cortex rather than the motor cortex. The localization of motor cortex helped facilitate the decision to operate and the planning of the surgery, in particular by defining the extent and the margins of the resection (see Figures 2A, 5 for examples). It also aided in educating parents regarding the planned surgery and providing reassurance that the motor function would be intact post-operatively.

TMS motor mapping is also useful in characterizing normal motor development in young children with neurological disorders. While TMS stimulation of the motor cortex usually elicited an MEP response in the contralateral muscles, we often observed responses in the ipsilateral muscles as well (see Figure 3A), indicative of the immature level of motor development in this age group. During normal development, crossed (contralateral) corticospinal tracts, projected to the spinal cord at birth, strengthen preferentially, and by 2 years of age, uncrossed (ipsilateral) tracts disappear (64). Our results indicate that similar motor developmental trajectory exists in many children with neurological diseases, as in this cohort, we did not observe MEPs in ipsilateral muscles in children older than 2 years with intact motor cortices.

However, we observed persistent ipsilateral MEPs in children with a history of perinatal injury leading to stroke. In such children, we observed robust representation of both upper extremities in the intact hemisphere (see Figure 4B). Such interhemispheric reorganization has previously been reported in children who had suffered perinatal brain injury (10, 20, 70, 74, 75), although the functional relevance of such interhemispheric reorganization is yet to be clearly understood. We also observed patterns of reorganization where the hand motor representation was noted in the putative leg area (See Figure 4A) or in the premotor cortex. This type of intrahemispheric reorganization was noted in children with cortical dysplasia. Since malformation of cortical development and neuronal migration disorder is observed in cortical dysplasia (76), the neurons destined to the hand motor cortex likely end up at aberrant locations in the same hemisphere. TMS is therefore a vital means to demonstrate the functionality of such dysplastic cortex, since the presence or absence of motor function in the lesion will influence the surgical decision.

### Language Mapping

The language cortices in temporal and frontal regions were successfully mapped by TMS in both hemispheres in nine pre-school aged children. Most frequently, TMS identified critical language areas in both hemispheres. Findings of the presence of language cortices in both hemispheres (often R>L) in these children is consistent with previous reports and provides insight into language organization in this age group. For instance, onset of epilepsy in young children has been shown to adversely affect language development (77, 78). Functional MRI studies have shown that many types of epilepsies alter the trajectory of maturation of language networks. Therefore, greater bilateral activation for language tasks is observed in children with epilepsy when compared to greater engagement of the left hemisphere in typically developing children (79–82). Thus, the bilateral representation and right hemisphere dominance for language in the study cohort, as identified by TMS, most likely represents a combination of ongoing development, false positives (discussed below), and the effects of refractory epilepsy or a brain tumor on the organization of language networks in this young cohort. Similar to our findings here, another recent TMS study has reported receptive language areas in the right hemisphere in children between 6 and 10 years of age with a brain tumor or epilepsy (83).

To date, few studies have compared the accuracy of TMS-derived language maps against regions identified by other invasive and non-invasive mapping methods. Studies comparing language localization by TMS and CSM have demonstrated TMS to have high sensitivity (63–97%), and a negative predictive value (74–99%), but with variable specificity (17–97%) stemming from high rates of false positive errors when compared to CSM (7, 13, 47, 48). But these studies are mainly in adults, and only a small number of children have been included (13, 47). In a small cohort of six patients (age range 14–37 years), we compared the HD estimated by TMS against that from Wada testing (45) and found the overall accuracy of TMS in identifying language in a hemisphere to be 79% with a diagnostic odds ratio of 14, indicating moderate agreement between the two modalities. In a primarily pediatric cohort, we have found that TMS-derived HD had high sensitivity and specificity with an overall accuracy of 80% when compared to non-invasive counterparts, MEG (49) and fMRI (45). Based on these reports, we expect the TMS-derived language mapping to have similar efficacy in this younger cohort as well. Only one other group has reported attempting speech and language mapping in children between 4 and 6 years of age, limited to the lesioned hemisphere (84, 85) and reporting a 40% success rate. Similar to our study, these researchers reduced the SO such that the children were comfortable with stimulation intensity used and the SO ranged from 24 to 36%, corresponding to an E-field of 39–66 V/m.

The findings from TMS language mapping in this cohort were used in surgical planning in seven children in whom the epileptogenic focus or brain tumor was in close proximity to the language cortices. In most cases, MEG and fMRI were unsuccessful, and even when successful, these modalities provided information on the receptive language areas only, as they were performed under sedation. The localization of the language cortex by TMS aided in the surgical decision to operate and in surgical planning, in particular by defining the extent and the margins of the resection. It also helped facilitate discussions with the family regarding the risks of language deficits and the likelihood of preserved language functions post-surgery. Consistent with our expectations, the seven patients had no deficits in language functions following surgery. Finally, TMS language mapping also provided a baseline with which to evaluate post-surgical changes in language function.

This study demonstrates the challenges of language mapping in young children. While, temporal lobe stimulation was well-tolerated, nearly half the children could not endure stimulation of frontal lobe language cortices, despite lower stimulation intensities used in this area. Since the stimulation intensity used for speech and language mapping was well below their UE motor threshold, it is very unlikely that speech errors observed during TMS were due to the stimulation of premotor, Broca's, or primary mouth/laryngeal motor cortices in the frontal lobe. More commonly, we found excessive stimulation of face and jaw muscles with frontal lobe TMS, which often caused discomfort, pain, and/or speech errors. Due to their young age, procedures used in adults to further delineate speech and language errors, such as neurophysiological recordings from laryngeal muscles (86, 87) or accelerometer-based voice onset detection (88), were not feasible. Therefore, we carefully reviewed the mapping session and discarded trials of speech arrest or hesitation observed with apparent discomfort or excessive muscle movement.

Patient compliance was another factor frequently affecting language mapping. Due to their young age, children often could not perform the task consistently, requiring frequent breaks and encouragement from the study team. The use of the color naming task was helpful in many of the children and improved their cooperation. Still, it was often challenging to differentiate TMS induced errors from baseline performance, likely leading to more false positive results than noted in older children and adults (45, 49). Moreover, the decreased compliance precluded extensive surveys of the temporal and especially frontal regions, and therefore, the findings might be biased due to incomplete sampling of critical language areas in this population. Another factor to be cognizant of is the anti-seizure medications taken by children. For example, topiramate and zonisamide have been shown to cause speech difficulties, including problems with word selection and slower response time, in children (89). The unpredictable nature of AED-induced apparent speech errors make analysis challenging both at baseline and during TMS. Finally, the influence of these patient parameters can only be fully deduced when TMS language mapping data in a comparable normative population is available. Despite these challenges, we believe meaningful information was provided by the TMS language mapping.

In addition to the aforementioned patient-related drawbacks, TMS parameters should also be taken into consideration to improve TMS language mapping in children. Key TMS parameters that affect language mapping results include task type, TMS onset relative to stimulus presentation, intensity, coil orientation, and rate. Our previous studies suggest that TMS intensities of 70–100 V/m independent of individual motor threshold (MT) successfully elicited speech errors while minimizing unsuccessful results from either patient discomfort due to too high an intensity or failure to elicit errors due to too low an intensity (45, 49). These findings support not basing TMS intensity on MT, as currently recommended (9), since MT is high in this age group. TMS applied at 100% of an already high resting MT can be painful due to muscle stimulation and is likely to result in false positive responses. Moreover, we did not find that the stimulation intensity differed across types of speech errors or between hemispheres in each individual. Although we used a color/object-naming task consistently across patients in TMS, data from reported fMRI studies indicate that this task results in bilateral and variable patterns of activation (90, 91), making it impossible to dissociate the effects of task-induced engagement of bilateral language cortices from language reorganization. Therefore, task choice should be prioritized for optimization, with verb generation as a particularly promising task for study, as it is child-friendly, is already implemented in fMRI (92–94), and is suitable for performing during TMS.

TMS inaccuracies also result from issues of non-reproducibility of speech errors and over-reliance on non-specific errors. In our patient cohort, non-specific performance errors were the most common type of error elicited by TMS. However, these errors are more difficult to correctly identify by raters because they can resemble errors made during baseline speech performance. It has been generally assumed that higher TMS rates result in greater disruption of the stimulated region, and consequently in an increased number of specific errors. We used a fixed TMS rate of 5 Hz in all patients, but recent studies have found that rates ≥10 Hz resulted in an increased number of speech errors (95), and at 7 Hz, a greater percentage of elicited errors were speech arrests with fewer hesitation errors (96). While these findings suggest that higher rates may be more effective at inducing reliable speech errors, examining these parameters in young children is difficult due to decreases in compliance over time and the need to balance intensity-related discomfort with efficacy. Moreover, in order to allow a wider survey of brain areas while keeping the total number of stimulation within safety guidelines, we fixed the TMS frequency to 5 Hz. Another factor that can influence the error type is the timing of TMS stimulation in relation to the stimulus onset. We time-locked TMS to stimulus onset to ensure adequate coverage of the occipitotemporal or ventral pathway of object recognition (97) and early language processing occurring in the temporal cortex (51). TMS delivered with no delay has also been shown to result in fewer false negative results when compared to CSM (52). However, these findings have been reported in adults, and similar data are not available for young children. Indeed, the visual-language pathways and language-related processes could be delayed and/or longer in young children (98, 99) and therefore the TMS timing relative to the stimulus may have to be adjusted accordingly. Future work should be directed at optimizing the different TMS parameters and developing mapping strategies aimed at improving the accuracy of TMS language mapping in children.

## Safety of TMS

In this study, TMS was safely applied in young children with serious epilepsy syndromes. The most common side effect was mild and included local pain and discomfort during language mapping. Motor mapping using single-pulse TMS was usually well-tolerated and experienced by most children as painless. About 20% of the motor mapping cohort experienced seizures during or immediately following TMS. However, the seizures were consistent with their typical semiology and were deemed to not be directly caused by TMS. The occurrence of TMS-related seizures in patients with epilepsy is a known complication (100, 101), and the presence of medically intractable epilepsy has been known to increase the likelihood of a typical seizure occurring during TMS (102). However, in all reports of a seizure during TMS, the patients had their typical seizure followed by their typical recovery course (100, 101). The crude seizure risk for an adult with epilepsy is estimated to be 2.9% (103) to 3.6% (101) for single-, paired-pulse, and rTMS protocols. In our cohort, we performed a total of 60 sessions, with a seizure occurring in 10 of these; though the TMS-related seizure rate appears to be higher than previous reports, it is important to note that unlike other studies, our cohort primarily consisted of young children, and that children have lower seizure thresholds than adults. Furthermore, many conditions identified as associated with increased TMS-induced seizure risk in the TMS safety guidelines (50) were present in this clinical cohort at the time of testing; the patients had refractory epilepsy, were often sleep-deprived and anxious due to stressors associated with inpatient hospitalization, and had temporarily reduced or discontinued their anti-seizure medications for monitoring. Additionally, because this cohort consists of children who, in many cases, were already experiencing multiple seizures per day, the likelihood of seizure while in the TMS exam room was relatively high, regardless of stimulation. For instance, some children had seizures during transportation to the TMS lab or during initial setup before any TMS was applied. By comparing each child's TMS-related seizure timing, semiology, duration, and recovery to the child's own typical seizure, we were able to determine that the majority of seizures occurring during the TMS exams in this cohort were unlikely to be TMS-induced, but rather represent the patient's characteristic seizure pattern. Furthermore, at our institution, all TMS studies are performed in the presence of a nurse with immediate access to rescue medication. None of the children who had a seizure during or following TMS required administration of oxygen or intravenous AEDs. Finally, children's subjective experience of TMS places it in the middle of a spectrum of ordinary childhood experiences (104). Therefore, all available data so far indicate that the use of TMS in children is safe. However, it is recommended that safety precautions be taken during a TMS study in children, including having medical personnel and rescue medications at the ready during mapping. The rate and intensity parameters of TMS should be within the International Federation of Clinical Neurophysiology (IFCN) guidelines for safety (50). Patients should be continuously monitored visually and by EMG for signs of seizures or intracortical spread of excitation.

## Limitations

This study does have some limitations. MEPs were elicited by applied TMS along the precentral gyrus, and due to the challenges of performing a systematic examination, complete motor mapping was not possible in these young children. However, eliciting MEPs using TMS stimulation confirmed the presence of motor areas along the precentral gyrus. Although three children had only one clear response in each hemisphere, most children had five or more clear MEPs and/or CSPs. The 14% average response rate in this cohort, which was well below the average incidence of responses typically used to define MT (i.e., 50%), reflects the high MT of these children, which likely exceeded maximum SO. As such, even a single clear response in each hemisphere, isolated from background EMG noise and spontaneous activity, was considered representative of the motor cortex. Improvements in coil design to deliver greater E-fields should be considered in future studies to increase the response rate in this population.

The 70 mm figure-of-eight coil used in this study stimulates a large area of cortex under the coil, especially at 100% SO. There is therefore a possibility that MEPs could result from stimulation of the cortical area not directly beneath the coil center, leading to mislocalization. However, in all our patients, at each stimulation site, MEPs were elicited from only one muscle group, indicating that the stimulated area was most likely small. Nevertheless, care should be taken to keep the stimulation more focused, especially when performing pre-operative mapping prior to resection of a dysplasia or cortical neoplasm. With respect to language mapping, all the critical language areas were not surveyed in this young cohort and it is possible that the TMS intensity was too low to elicit reliable speech errors. It is also possible that the still-developing language networks in this young cohort may be less susceptible to lesioning and/or require a higher TMS intensity. The tradeoff between higher TMS intensity and pain during stimulation should be considered on an individual basis. Finally, the study lacks a direct comparison against other invasive and non-invasive mapping methods with respect to its efficacy in presurgical mapping or in predicting postoperative function. Future prospective studies should be designed to address this drawback.

## Conclusions

In summary, we demonstrate the feasibility of using TMS to directly localize the motor, speech, and language systems without using conscious sedation and its utility in presurgical planning in a cohort of young children. We also provide evidence that TMS is well-suited to probe motor, speech, and language pathophysiology and plasticity in young children. Specifically, our data show that TMS can be a useful tool in mapping eloquent cortices in children with epilepsy or brain tumor, both on and off AEDs. Our experience indicates that TMS-derived motor and language maps are helpful in surgical planning, educating parents regarding the planned surgery, and providing a baseline to evaluate post-surgical changes in motor and language functions. Future large-scale studies are needed to confirm the effectiveness and reliability of TMS language mapping in this population.

## Data Availability Statement

The data analyzed in this study is subject to the following licenses/restrictions: Clinical data with patient HIPPA information cannot be shared with investigators outside of the institution. Requests to access these datasets should be directed to snaraya2@uthsc.edu.

## Ethics Statement

The studies involving human participants were reviewed and approved by Institutional Regulatory Board, University of Tennessee Health Science Center, Memphis TN. Written informed consent to participate in this study was provided by the participants' legal guardian/next of kin. Written informed consent was obtained from the individual(s), and minor(s)' legal guardian/next of kin, for the publication of any potentially identifiable images or data included in this article.

## Author Contributions

SN and SG were responsible for the design and conceptualization of the study. SN oversaw the acquisition, analysis, and interpretation of the data and drafted and revised the manuscript. SG acquired and processed TMS data, assimilated clinical and imaging data, and revised the manuscript. SF, AM, BM, SW, FB, and JW oversaw the clinical care of patients and interpreted the clinical and imaging data. All authors provided critical revision of the manuscript for important intellectual content.

## Conflict of Interest

The authors declare that the research was conducted in the absence of any commercial or financial relationships that could be construed as a potential conflict of interest.

## Abbreviations

AED: Antiepileptic drugs
APB: Adductor Pollicis Brevis
CSM: Cortical Stimulation Mapping
E-field: Electric Field
EEG: Electroencephalogram
EMG: Electromyography
fMRI: Functional MRI
FSPGR: Fast Spoiled Gradient-echo
GABA: γ-aminobutyric acid
HD: Hemispheric Dominance
LE: Lower Extremity
LH: Left Hemisphere
LI: Laterality Index
MEG: Magnetoencephalography
MEP: Motor Evoked Potentials
MRI: Magnetic Resonance Imaging
MT: Motor Threshold
RH: Right Hemisphere
SD: Standard Deviation
SO: Stimulator Output
TA: Tibialis Anterior
TMS: Transcranial Magnetic Stimulation; MRI-guided TMS
TSC-2: Tuberous Sclerosis Complex type 2
UE: Upper Extremity
VAS: Visual Analog Scale.
